# Protective Effects of *Myrtus communis* Essential Oil Against Bisphenol A-Induced Sperm Dysfunction: Insights from Lipidomic, Amino Acid Profiling, Oxidative Stress and Molecular Docking

**DOI:** 10.3390/antiox15050536

**Published:** 2026-04-24

**Authors:** Mariem Mhimdi, Slimen Selmi, Wael Taamalli, Stefania Sut, Hichem Sebai, Stefano Dall’acqua

**Affiliations:** 1 Laboratory of Functional Physiology and Valorization of Bio-Resources, Higher Institute of Biotechnology of Beja, University of Jendouba, Beja 9000, Tunisia; slimen.selmi@isbb.rnu.tn (S.S.); hichem.sebai@u-jendouba.tn (H.S.); 2 Laboratory of Olive Biotechnology, Center of Biotechnology of Borj Cedria, P.O. Box 901, Hammam Lif 2050, Tunisia; wael.taamalli@cbbc.rnrt.tn; 3 Department of Pharmaceutical and Pharmacological Sciences, University of Padova, Via Marzolo 5, 35131 Padova, Italy; stefania.sut@unipd.it (S.S.); stefano.dallacqua@unipd.it (S.D.)

**Keywords:** bisphenol A, lipidomic, molecular docking, *Myrtus communis* essential oil, oxidative stress, sperm toxicity

## Abstract

BisphenolA (BPA) is a common endocrine disruptor that impairs male fertility through oxidative stress and alterations in membrane lipids. This study evaluated the protective effects of *Myrtus communis* L. essential oil (EOMC) on BPA-induced sperm toxicity in Wistar rats in vitro. BPA significantly decreased sperm motility and viability. It also increased lipid peroxidation, depleted thiols, and reduced the activity of antioxidant enzymes (SOD, CAT-like and GPx-like). Concomitant treatment with low and intermediate doses of EOMC (0.5–1 µL/mL) restored sperm function, reduced oxidative stress, and preserved membrane phospholipids. However, the highest dose (5 µL/mL) further impaired sperm function and disrupted membrane phospholipids. BPA also altered amino acid profiles and accumulated intracellularly, effects partially reversed by EOMC, which redistributed free BPA into the culture medium. Bioavailability analysis revealed selective absorption of α-pinene, while d-limonene and 1,8-cineole were undetectable. Molecular modeling indicated strong binding of BPA to antioxidant enzymes, potentially disrupting their structure and activity. Overall, these results show that EOMC protects sperm from BPA-induced damage in a dose-dependent manner through antioxidant, membrane-stabilizing, and redistribution mechanisms. This highlights its potential application in phytotherapy for male reproductive health.

## 1. Introduction

Male fertility is increasingly threatened by exposure to endocrine disruptors, among which bisphenol A (BPA), also known as 2,2-bis (4-hydroxyphenyl) propane, is a monomer commonly used to produce polycarbonate plastics and epoxy resins [1]. It is widely utilized in industries such as plastics manufacturing, food packaging, and thermal paper production [2,3]. However, research has shown that BPA acts as an endocrine disruptor, interfering with hormonal mechanisms and affecting reproductive health [4,5]. In particular, BPA has been shown to impair sperm human motility [6] and viability [7], damage DNA, and induce oxidative stress [8]. In addition, we also reported that a BPA-induced increase in ROS levels in spermatozoa was associated with a premature acrosome reaction, ionic imbalance, alterations of the sperm proteome [9] and intracellular adenosine triphosphate (ATP) content [10]. On the other hand, in vitro studies in animal models showed that direct exposure to BPA can negatively affect the motility of spermatozoa [10,11].

In view of these concerns, it is crucial to seek therapeutic strategies to counter the toxic effects of BPA on fertility. Recently, a growing interest has been drawn to the use of traditional medicinal plants for the treatment of human diseases and, in particular, infertility and reproductive toxicity [12].

The essential oil of *Myrtus communis* L., derived from myrtle, is renowned for its antioxidant [13], antibacterial [14], and anti-inflammatory properties [15]. *Myrtus communis* is known for its protective potential against damage induced by environmental toxins [16]. It has also been widely used in the pharmaceutical and food industries due to its high content of bioactive ingredients [17].

Importantly, this in vitro study is designed to strategically complement our group’s previously published in vivo work on BPA-induced reproductive toxicity [18,19], allowing a more detailed exploration of sperm motility, viability, oxidative stress, membrane phospholipids, amino acids, and BPA bioavailability. This approach provides mechanistic insights that support and extend our earlier in vivo findings.

The aim of this study was to quantify BPA metabolites in spermatozoa and a culture medium, assess the effects of BPA on sperm motility and viability, and examine the protective effects of *Myrtus communis* L. essential oil. In addition, sperm membrane phospholipids, amino acid profiles, oxidative stress markers, and the bioavailability of myrtle active compounds were analyzed.

## 2. Material and Methods

### 2.1. Plant Collection

Myrtle leaves were harvested in March in the Hammam Bourguiba region (in the north-west of Tunisia) and identified by the botanist Chokri Hafsi.

### 2.2. Essential Oil Preparation

The extraction method for the essential oil from *Myrtus communis* leaves was hydrodistillation for 3 h using a Clevenger-type apparatus. In summary, the plant material was submerged in water and brought to a boil, causing the essential oils to evaporate along with the water vapor. The vapors were then condensed and collected. The distillate was subsequently isolated and dried over anhydrous sodium sulfate. The oil fractions were stored at 4 °C until use [20].

### 2.3. GC–MS Characterization of Myrtus communis Essential Oil

The chemical composition of *Myrtus communis* essential oil used in this study was previously characterized by GC–MS in our earlier work. The oil was obtained from the same botanical source and prepared under identical conditions as previously described [21]. The major constituents were α-pinene (59.749%), 1,8-cineole (18.651%) and d-limonene (7.020%). The full GC–MS profile of the oil batch is provided in Supplementary Table S1.

### 2.4. Sperm Processing and Design

#### 2.4.1. Animals

Fifteen male Wistar rats, aged 7 to 8 weeks and weighing between 200 and 250 g, were obtained from a pet store affiliated with the Higher Institute of Biotechnology of Beja. All experimental procedures involving animals were approved by the Institutional Animal Ethics Commission of the University of Jendouba, Tunisia (Approval No. UJ2025-018-0061; Project ID: UJ-ISBB (LPFVBR)11/2025; approval date: 22 September 2025) and were conducted in accordance with institutional guidelines for the care and use of laboratory animals. Rats were randomly assigned to five experimental groups (*n* = 3 per group). Each assay was performed in triplicate (3 technical replicates) for each biological sample. Animals were kept under standard pet shop conditions (22 ± 0.5 °C, and 12 h/12 h light/dark cycle), with access to food (standard pellet diet- Badr Utique-TN) and water ad libitum.

#### 2.4.2. Collection and Incubation of Epididymal Spermatozoa

Spermatozoa were collected from the fresh epididymides of adult rats according to the method of Fouad, El-Dakdoky et al. [22]. Briefly, the epididymis was dissected into 4–5 sections using a sharp razor blade and then placed in a modified Ringer’s phosphate solution (RPS) containing NaCl (119 mM), KCl (5 mM), MgSO_4_ (1.2 mM), glucose (10 mM), potassium phosphate (16.3 mM), and penicillin (50 units/mL), with a pH of 6.9. The sperm suspension was adjusted to a final concentration of 10 × 10^6^ spermatozoa/mL and exposed to in vitro treatments at 37 °C under different conditions.

#### 2.4.3. Experimental Design and Treatments

Three experimental conditions were established to evaluate the effects of BPA and *Myrtus communis* essential oil (EOMC) on rat spermatozoa.

Condition 1 (BPA exposure): Spermatozoa were incubated for 1 h in the presence of BPA at increasing concentrations of 10, 50, 100, 200 and 300 µM, to assess dose-dependent cytotoxicity.

Condition 2 (co treatment): Condition 1 protocol was applied:

First, spermatozoa were incubated for 1 h with different concentrations EOMC (0.5, 1 et 5 μL/mL); Then, a co-incubation of 1 h was performed with the maximum toxic dose of BPA (as identified in Condition 1), to evaluate potential protective effects of EOMC.

In all experimental conditions, a corresponding control group was included, incubated under identical conditions without exposure to either EOMC or BPA.

#### 2.4.4. Evaluation of Sperm Motility and Viability

Ten-microliter samples of sperm were transferred onto a pre-warmed microscope slide and examined under a light microscope at 400× magnification. Sperm motility was evaluated visually according to the criteria described in the laboratory manual of the World Health Organization [23]. Spermatozoa were classified into three categories: progressive motile (PR), non-progressive motile (NP), and immotile (IM). Progressive motility was defined as sperm moving actively forward in a straight line or large circles, whereas non-progressive motility referred to sperm showing movement without forward progression. Immotile sperm showed no movement. The proportion of motile sperm in each observed field was calculated by dividing the number of motile sperm by the total sperm count, and the average across all fields was then determined. Sperm motility was presented as the percentage of motile sperm relative to the total sperm count.

Sperm viability was assessed using the eosin–nigrosin staining technique, following WHO guidelines [24]. In brief, 10 µL of each sperm sample was combined with 10 µL of eosin–nigrosin stain (Sigma, St. Louis, MO, USA) and left at room temperature for 30 s. The mixture was then examined under a light microscope. Non-viable sperm displayed red-stained heads, whereas viable sperm remained unstained and appeared bright. Sperm viability was expressed as the percentage of intact cells.

### 2.5. Extraction and Quantitative Determination of Lipid Sperm Cells by LC-MS

For the LC-QTOF analysis, a chromatograph Waters Acquity UPLC was used, coupled with a Waters Xevo G2 Quadrupole Time of Flight (QTOF) mass spectrometric (MS) detector (Waters Corporation, Milford, MA, USA). In stationary phase, an Acquity Premiere BEH HILIC (2.1 × 100 mm, 1.7 µm, Waters Corporation, Milford, MA, USA) column was used, and column temperature was maintained at 40 °C. A mixture of water + 0.1% formic acid (A) and Acetonitrile + 0.1% formic acid (B) was used as the mobile phase. The elution gradient was as follows: 0–1 min, 10% A; 15 min, 25% A; and 18 min, 30% A. Flow rate was 0.2 mL/min, and the injection volume was 1 μL. MS data were acquired in positive ionization mode (ESI+) in the mass range 50–2000 Da. The sampling cone voltage was adjusted at 40 V, the source offset at 80 V. The capillary voltage was adjusted to 3.5 KV. The nebulizer gas used was N_2_ at a flow rate of 800 L/h. The desolvation temperature was 450 °C. The mass accuracy and reproducibility were maintained by infusing lock mass through Lockspray at a flow rate of 20 μL/min. The *m*/*z* value of all acquired spectra was automatically corrected during acquisition based on lock mass. A MS^E^ experiment was simultaneously performed to collect structural information, setting the collision energy to 30 V. Compounds were identified based on HR-MS and MS^E^ fragmentation.

### 2.6. Sperm Cell and Culture Medium Preparation and Evaluation of Amino Acids by LC-MS

#### 2.6.1. Sperm Amino Acid Extraction

Semen metabolomic analysis was performed according to [25], with some modifications. Briefly, epididymal sperm pellets were first resuspended in 2 mL of methanol/water (1:1, *v*/*v*) and homogenized by ultrasonication for 2 min on ice to ensure complete cell lysis. The homogenates were then centrifuged at 5300× *g* for 5 min at 4 °C, and the resulting supernatants, containing extracted metabolites, were carefully collected. For further extraction and cleanup, 300 µL of 5 mM ammonium acetate in methanol was added to each supernatant to extract metabolites and internal standards. The mixtures were shaken at 450 rpm for 30 min at room temperature using a Thermomixer Comfort (Eppendorf, Hamburg, Germany), followed by centrifugation through a solid-phase filtration membrane to remove particulates. Prior to injection, the final extracts were diluted if necessary and analyzed by LC-MS/MS using an injection volume of 10 µL.

#### 2.6.2. LC-MS/MS Analysis

An Agilent LC 1200 (Agilent, Milford, MA, USA) series liquid chromatography system with a photodiode array detector (HPLCPDAD) and an AB/Sciex API 3200 QTRAP LC/MS/MSn mass spectrometer (AB Sciex, Framingham, MA, USA) comprise this system. HPLC studies were carried out on a C18 column (250 × 4.6 mm, 3.5 m, Kherad Azma, Tehran, Iran), the solvent system was set to 1 mL/ min, and the solvent composition and elution followed a linear gradient of H_2_O: ACN for 60 min, from 90% H_2_O to 10% ACN. The sheath gas flow rate was 60 mL/min, the auxiliary gas flow rate was 20 mL/min, the spray voltage was 4.5 kV, the capillary temperature was 200 °C, the capillary voltage was 46 kV, and the tube lens was −60 kV. The mass range was set at *m*/*z* 45–1000, with a scan speed of 2400 atomic mass unit (amu) per sec. To replicate product ions, several combinations of collision energy were utilized. The automatic selected reaction monitoring (SRM) mode optimization was employed to establish appropriate collision energies in the range of −5 to −180 V once stable product ions were produced.

### 2.7. Sperm Cell and Culture Medium Preparation and Evaluation of Free Bisphenol A by LC-MS

#### 2.7.1. Extraction of BPA from Sperm Cells

Extraction of free bisphenol A is performed by liquid–liquid extraction (LLE), according to the method described by [26], with some modifications. Briefly, after thawing at room temperature, 0.2 mL of sperm culture medium or sperm pellet was transferred into a 15 mL polypropylene tube, and 744 ng/mL of benzanilide. The sample was extracted three times, each with 2 mL of ethyl acetate. The extracts were combined, washed with milli-Q water, concentrated to near-dryness under N2, and reconstituted with 0.2 mL of methanol before injection into LC–MS/MS.

#### 2.7.2. LC-MS/MS Analysis

The quantitative analysis of free bisphenol A was carried out using a high-performance liquid chromatography system coupled with a triple quadrupole tandem mass spectrometer (LC-MS/MS), consisting of a Varian binary pump, autosampler, diode array detector, and a triple quadrupole MS320 (Varian/Agilent Technologies, Santa Clara, CA, USA). Electrospray ionization (ESI) was performed in negative ion mode, using the multiple reaction monitoring (MRM) acquisition mode. Chromatographic separation was achieved using an Agilent XDB C18 column (3 × 150 mm, 3.5 µm) maintained at 35 °C. The mobile phase consisted of two solvents: eluent A (water with 0.1% formic acid) and eluent B (methanol with 0.1% formic acid). The elution gradient started with 90% A (10% B) held for 0.5 min, then shifted to 50% A/50% B, reaching 100% B at 10 min, and maintained isocratically until 20 min. The column was re-equilibrated to 90% A from 20 to 25 min. The flow rate was set at 0.3 mL/min, and the injection volume was 10–20 µL depending on the sample.

### 2.8. Sperm Cell and Culture Medium Preparation and Analysis of Bioavailability Compounds from EOMC by GC-MS

#### 2.8.1. Extraction of Compounds

This protocol was adapted from [27], with minor modifications. Briefly, active compounds from EOMC were extracted from spermatozoa and their culture medium using a liquid–liquid extraction method, followed by analysis via gas chromatography–mass spectrometry (GC-MS). Sperm cells (pellet) and 100 µL culture medium (supernatant) were transferred into clean 1.5 mL Eppendorf tubes, followed by the addition of 100 µL of a solvent mixture composed of hexane and ethyl acetate (1:1, *v*/*v*). The mixture was vortexed vigorously for 1 min to facilitate the extraction of lipophilic compounds. The samples were then centrifuged at 12,000 rpm for 10 min at low temperature using a cryogenic high-speed centrifuge to prevent degradation of thermosensitive compounds. After phase separation, the upper organic layer containing the extracted compounds was carefully collected using a micropipette and transferred into clean 1.5 mL autosampler tubes. Finally, 1 µL of the organic extract was injected into a gas chromatography–mass spectrometry (GC-MS) system for analysis.

#### 2.8.2. GC-MS Analysis

For the analysis, a Varian 3800 gas chromatograph (Varian, Palo Alto, CA, USA) was used, coupled with Saturn 2000 T MS mass spectrometer (Varian, Palo Alto, CA, USA) using EI as ionization source. For quantitative purposes, the mass spectrometer was set to work in MS/MS mode selecting *m*/*z* 91 as a precursor for monoterpenes and sesquiterpenoids. The selected ion was the *m*/*z* 231. The GC–MS/MS method allowed the identification and quantification of the EOs main constituents. The injector temperature was 225 °C—the oven started at 55 °C—and stayed isothermal for 5.5 min, then increased to 250 °C at 4 °C/min and then to 280 °C at 11 °C/min.

### 2.9. Evaluation of Biochemical Markers of Oxidative Stress

#### 2.9.1. Protein Assay

The protein content was measured according to [28]. This method is based on the ability of the protein–copper complex to reduce Folin’s reagent, resulting in a blue color at 650 nm. After constructing the calibration curve (optical density versus protein quantity in µg), the protein concentration of the assays was determined by plotting the optical density against the calibration curve using bovine serum albumin as a standard.

#### 2.9.2. Oxidative Stress Assessment

Malondialdehyde (MDA) levels were measured following the method outlined by Rhouma, Bahri et al. [29], which involves the reaction of MDA with thiobarbituric acid. Thiol group (-SH) estimation was conducted in accordance with Bergsma, Li et al.’s method [30], whereas SOD activity was assessed following the procedure described by Misra and Fridovich, utilizing the epinephrine/adrenochrome system [31]. The protocol outlined by Garrido, Meseguer et al. was employed to examine Glutathione peroxidase (GPx) activity [32], and catalase (CAT) activity was determined using the method described by Hadwan [33].

### 2.10. In Silico Molecular Docking

#### 2.10.1. Protein Structure Modeling and Validation

Amino acid sequences for three antioxidant enzymes from Rattus norvegicus were retrieved from the UniProt knowledgebase: Catalase (UniProt ID: P04762), Cu/Zn Superoxide Dismutase (SOD1, P07632) and Glutathione Peroxidase 1 (GPX1, P04041) [34]. Three-dimensional structures of the antioxidant enzymes were generated using AlphaFold3 via the AlphaFold Server [35]. The predicted local distance difference test (pLDDT) scores were examined to assess model confidence, with all models showing pLDDT > 90 for >90% of residues, indicating very high prediction confidence. Oligomeric assemblies were constructed by structurally aligning these monomers onto experimental templates using the Superposition tool in Maestro [36]. The tetrameric assembly of catalase was modeled using bovine liver catalase (PDB: 7CAT) as a template, the GPX1 tetramer was modeled using human GPX1 (PDB: 2OBA) and the SOD1 dimer was modeled using human SOD1 (PDB: 3JTT). Essential cofactors were incorporated using Maestro: the heme prosthetic group for catalase, Cu^2+^ and Zn^2+^ ions for SOD and the selenocysteine residue (Sec47) for GPX1. Structural validation was performed to ensure model reliability using multiple complementary methods. The stereochemical quality of each enzyme model was assessed using the SAVES v6.1 server (https://saves.mbi.ucla.edu/). Ramachandran plot analysis via PROCHECK [37] showed >90% residues in favored regions, with no residues in disallowed regions for SOD1 and GPX1 and only one residue in disallowed regions for catalase (Figure S1). Overall fold quality was evaluated using PROSA-web [38], with z-scores of −9.63 (catalase), −6.95 (SOD1) and −6.21 (GPX1), all falling within the characteristic range for native proteins of similar size. Local model quality was further assessed by plotting knowledge-based energy values against sequence positions, confirming that negative or near-zero energy values indicated error-free regions of the structures (Figure S1). Finally, each structure was prepared using the Protein Preparation Wizard [39]. Bond orders were assigned, hydrogen atoms were added and protonation states were assigned at physiological pH (7.0 ± 2.0) using Epik (implemented in Schrödinger Suite 2025-3) [40,41]. A restrained energy minimization was performed using the OPLS4 force field [42].

#### 2.10.2. Binding Site Detection and Receptor Grid Generation

Potential ligand-binding sites on each enzyme structure were systematically identified using SiteMap (Schrödinger Suite, Release 2025-4) [43,44] with default parameters. Sites were ranked by SiteScore. For each enzyme, top-predicted pockets were initially catalogued; many sites existed as symmetrical-related pairs or quartets due to the oligomeric nature of the enzymes. Representative sites were selected for focused docking: Sites 3, 4, 14, 15 and 17 for catalase; Sites 1–4 for SOD1; and Sites 1, 4, 7 and 11 for GPX1. SiteScores for selected sites are presented in Table 1. Receptor grids for molecular docking were generated using the Receptor Grid Generation panel in Glide [45,46,47]. For enzyme binding sites, the grid center was positioned at the centroid of the SiteMap-predicted pocket. The receptor grids were centered on the predicted pockets with a 20 Å × 20 Å × 20 Å box. Default grid generation parameters were used, including van der Waals radius scaling factor of 1.0 and partial charge cutoff of 0.25.

#### 2.10.3. Ligand Preparation, Molecular Docking and Binding Free Energy Calculations

SMILES representation of Bisphenol A was obtained from PubChem (BPA, CID: 6623). Ligand preparation was performed using LigPrep (Schrödinger, LLC. (2024). LigPrep, Release 2024-1, New York, NY, USA) [48]. For each compound, possible ionization states at pH 7.0 ± 2.0 were generated using Epik [40,49]. Low-energy 3D conformations were generated, and geometries were optimized using the OPLS4 force field [42]. Molecular docking of BPA was executed using Glide XP mode. For each binding site, up to 3 poses per ligand were retained based on Glide Score. To estimate binding affinities, the MM/GBSA (Molecular Mechanics with Generalized Born and Surface Area solvation) method was employed using the Prime module [50,51] for the top-scoring pose from each site. The binding free energy (ΔG_bind_) was calculated using the VSGB 2.0 solvation model [52] and the OPLS4 force field [42].

### 2.11. Statistical Analysis

All results were presented as mean ± SEM. Statistical analysis was performed using one-way analysis of variance (ANOVA) with GraphPad Prism statistical software, Version 9.0.2 (GraphPad Software Inc., La Jolla, CA, USA), to compare between groups. When significant differences were found, post hoc comparisons were performed using Tukey’s HSD to correct for multiple testing. *p*-values less than 0.05 were considered statistically significant.

## 3. Results

### 3.1. Effects of BPA and EOMC on Sperm Viability and Motility

**Figure 1:**
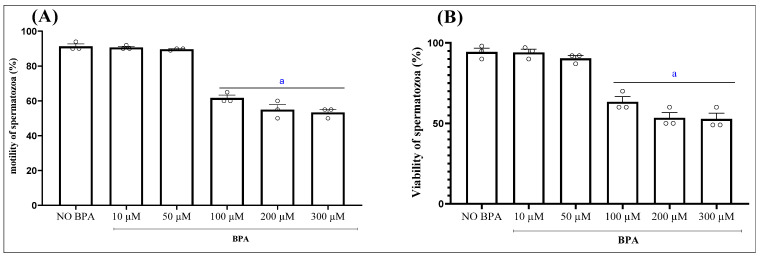
Effect of the incubation of epididymal sperm cells with bisphenol A (BPA), at different concentrations for 1 h, on sperm motility (**A**) and sperm viability (**B**). Results are reported, respectively, as percentage of viable and motile cells. Values are the mean ± SEM. Significance: ^a^ *p* < 0.05 compared to control group (ANOVA test). *n* = 3 independent biological replicates per group, with 3 technical replicates per assay.

In a series of preliminary trials, we evaluated the effect of one-hour exposure to various BPA concentrations, ranging from 10 μM to 300 μM, on sperm motility and viability (Figure 1A,B). Concentrations of 50 μM or below did not cause any significant changes in these parameters. However, from 100 μM onwards, a noticeable decrease in cell viability and a gradual decline in motility were observed, reaching a threshold beyond which no further reduction occurred. Based on these findings, we selected a BPA concentration of 100 μM for subsequent experiments.

**Figure 2:**
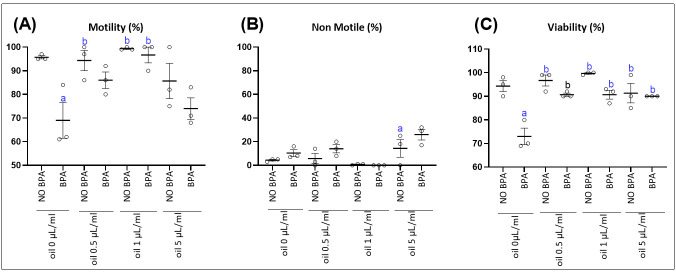
Effect of the incubation of epididymal sperm cells with EOMC for 1 h at different concentrations, followed by co-incubation for further 1 h with BPA, on sperm motility. (**A**) Sperm motility (**B**) and sperm viability. (**C**) Results are reported, respectively, as percentage. Values are the mean ± SEM. Significance: ^a^ *p* < 0.05 compared to control group and ^b^ *p* < 0.05 compared to BPA group (ANOVA test). *n* = 3 independent biological replicates per group, with 3 technical replicates per assay.

In the second set of experiments, spermatozoa were exposed to EOMC at various concentrations (0.5 μL/mL,1 μL/mL and 5 μL/mL) for one hour, followed by co-incubation with 100 μM BPA for another hour. The impact of these treatments on sperm parameters was then analyzed. However, co-incubation of EOMC at the lowest concentration tested (0.5 µL/mL) promoted a recovery of sperm motility and viability, reaching a level comparable to that of the control group (NO BPA). At 1 µL/mL, an almost complete restoration of sperm parameters was observed. In contrast, increasing the EOMC concentration up to 5 µL/mL led to a gradual decline in all these parameters (Figure 2).

### 3.2. Metabolomic Profiling of Lipid Composition in Sperm Cells In Vitro: Effects of BPA and Protective Treatments with Essential Oils

**Figure 3:**
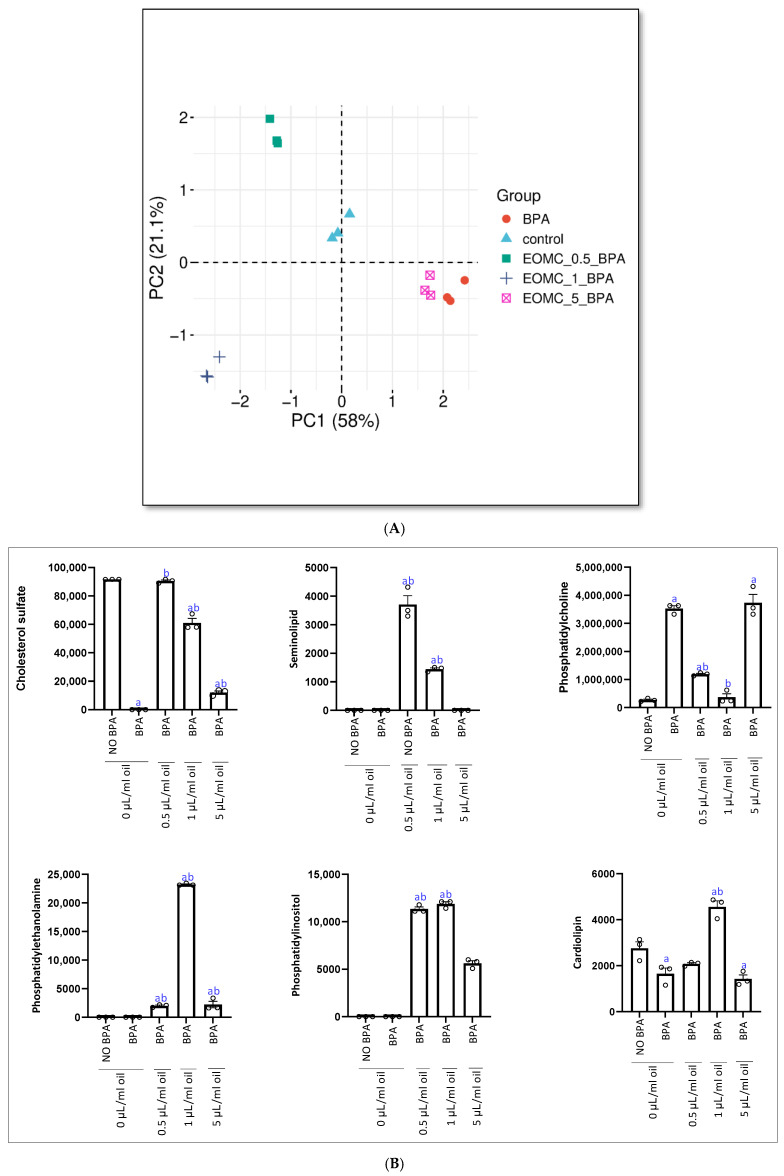
(**A**): Principal component analysis (PCA) score plot showing the distribution of different groups. (**B**): Effect of the incubation of epididymal sperm cells with EOMC for 1 h at different concentrations, followed by co-incubation for further 1 h with BPA, on lipid composition. Concentrations of key phospholipids. Values are the mean ± SEM. Significance: ^a^ *p* < 0.05 compared to control group and ^b^ *p* < 0.05 compared to BPA group (ANOVA test). *n* = 3 independent biological replicates per group, with 3 technical replicates per assay.

The elaboration was then performed using SRPLOT and different multivariate models were obtained (Figure 3A). At first, the PCA score plot shows a clear separation between the experimental groups, indicating distinct effects of the treatments on the analyzed data. The first principal component (PC1), explaining 58% of the variance, is the main axis of discrimination, separating the BPA and EOMC_5_BA groups on the positive side from the control and lower-dose EOMC groups on the negative side. This suggests that high-dose EOMC (5 µL/mL) induces a profile similar to BPA. The second component (PC2), accounting for 21.1% of the variance, further distinguishes the groups, particularly separating EOMC_1_BA, which appears to exhibit a unique response. The control group remains clustered near the center, while all treated groups show distinct shifts, confirming that both BPA and EOMC treatments significantly alter the overall profile in a dose-dependent manner.

Quantitative analysis of sperm membrane lipids revealed a variation in lipid composition between the different groups (Figure 3B). A significant increase in phosphatidylcholine was observed in the BPA-treated group compared to the control group (NO BPA). Incubation with *Myrtus communis* essential oil (EOMC) at 0.5 and 1 µL/mL induced a significant and dose-dependent decrease in this phospholipid. However, at a dose of 5 µL/mL, a tendency towards an increase in phosphatidylcholine was observed. Interestingly, BPA intoxication is associated with a decrease in cardiolipin, phosphatidylethanolamine, phosphatidylinositol, and seminolipid. However, co-incubation with 0.5 µL/mL EOMC tends to restore these levels, although the most marked effect is observed with the intermediate dose of 1 µL/mL. In contrast, at 5 µL/mL, a further decrease in these lipids is observed. Notably, the 0.5 µL/mL dose of EOMC appeared to prevent cholesterol sulfate and seminolipid depletion.

### 3.3. Effect of EOMC and BPA on the Amino Acid Composition of Spermatozoa

The analysis of amino acid profiles revealed significant alterations among the control group, the group exposed to BPA, and the groups co-treated with essential oil of *Myrtus communis* L. (EOMC) at concentrations of 0.5, 1, and 5 µL/mL (Table 2).

Exposure to BPA alone resulted in the complete disappearance of several amino acids, including arginine, histidine, phenylalanine, serine, and lysine, whose concentrations became undetectable relative to the control group. Concomitant treatment with EOMC at 0.5 and 1 µL/mL resulted in a partial restoration of the concentrations of some amino acids. However, at the highest EOMC dose (5 µL/mL), most of the amino acids whose concentrations had been restored at the lower doses became undetectable again (e.g., alanine, phenylalanine, and lysine), suggesting a potential biphasic response or toxicity effect at high concentrations. It is noteworthy that several amino acids, including methionine, glutamine, tryptophan, threonine, tyrosine, and asparagine, remain undetectable in all groups.

### 3.4. Effect of EOMC and BPA on BPA Free in Spermatozoa and Culture Medium

**Figure 4:**
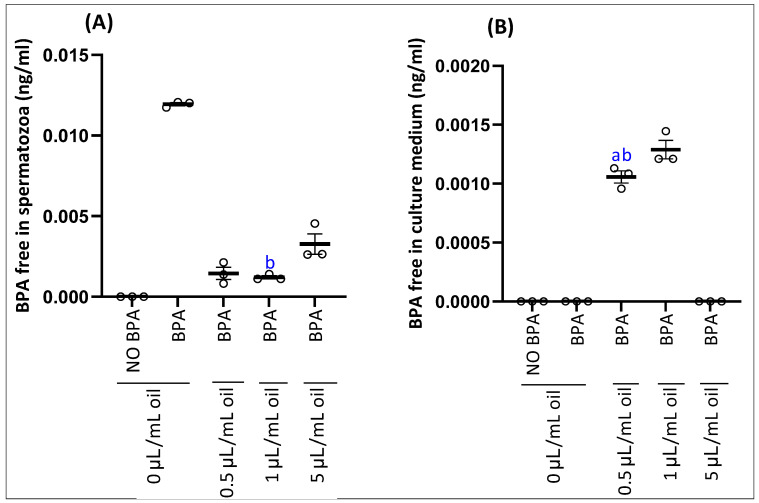
Effect of the incubation of epididymal sperm cells with EOMC for 1 h at different concentrations, followed by co-incubation for further 1 h with BPA on accumulation of BPA free on spermatozoa (**A**) and culture medium (**B**). Values are the mean ± SEM. Significance: ^a^ *p* < 0.05 compared to control group and ^b^ *p* < 0.05 compared to BPA group (ANOVA test). *n* = 3 independent biological replicates per group, with 3 technical replicates per assay.

Analysis of free bisphenol A (BPA) in spermatozoa (A) and their culture medium (B) is shown in Figure 4. Spermatozoa exposed only to BPA showed an accumulation of free BPA compared to the control (unexposed) group, while free BPA was absent in the culture medium. The addition of myrtle essential oil at concentrations of 0.5 and 1 µL/mL resulted in a significant reduction in the concentration of free BPA in spermatozoa, and this concentration was even lower at 5 µL/mL, with an accumulation of free BPA in the culture medium.

### 3.5. Bioavailability of Bioactive Molecules from Myrtle Essential Oil in Spermatozoa and Culture Medium

**Figure 5:**
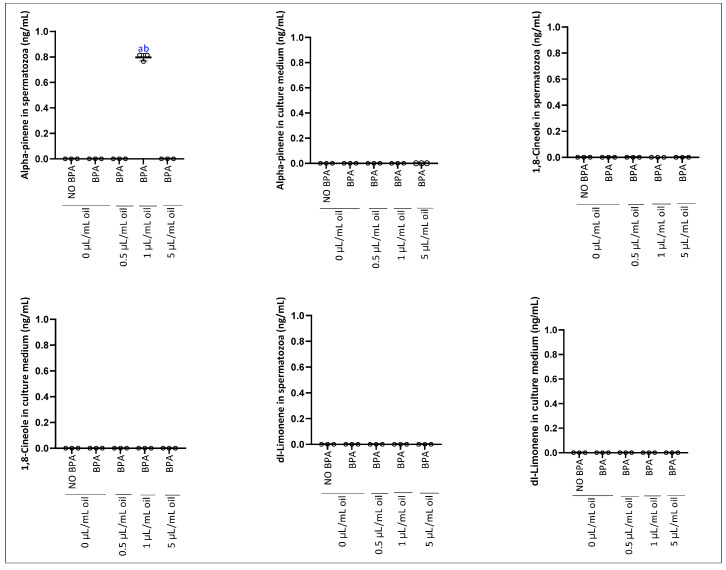
Effect of the incubation of epididymal sperm cells with EOMC for 1 h at different concentrations, followed by co-incubation for further 1 h with BPA, on the bioavailability of EOMC bioactive molecule spermatozoa and culture medium. Values are the mean ± SEM. Significance: ^a^ *p* < 0.05 compared to control group and ^b^ *p* < 0.05 compared to BPA group (ANOVA test). *n* = 3 independent biological replicates per group, with 3 technical replicates per assay.

The analysis of the bioavailability of bioactive molecules from EOMC in spermatozoa and the culture medium showed selective absorption depending on the administered dose. α-pinene was significantly detected only in spermatozoa at a concentration of 1 µL/mL, being absent at 0.5 and 5 µL/mL, as well as in the culture medium at all doses. In contrast, d-limonene and 1,8- were not detected in either spermatozoa or the culture medium (Figure 5).

### 3.6. Effect of BPA and EOMC on Sperm Oxidative Stress

#### 3.6.1. Lipid Peroxidation

BPA-induced lipid peroxidation at the cellular level, demonstrated by a significant increase in MDA levels compared to the control group (Table 3). EOMC treatment significantly protected against lipoperoxidation of cell membranes.

#### 3.6.2. Enzymatic Antioxidants

We found that BPA exhibited a significant decrease in the activity of antioxidant enzymes such as SOD, CAT-like, and GPx-like (Table 3) compared with the control group. EOMC treatment significantly restored the activity of these enzymes. It should be noted that the measured GPx and CAT activities correspond to GPx-like and CAT-like activities, as these assays may also reflect the contribution of other antioxidant systems present in spermatozoa.

#### 3.6.3. Non-Enzymatic Antioxidant

In the same context, (Table 3) showed that BPA induced a significant decrease in the level’s thiols compared with the control group.

### 3.7. Predicted Binding Sites and Interaction Profiles of BPA with CAT, SOD and GPX

Molecular docking simulations predicted high-affinity binding of BPA to multiple sites across rat antioxidant enzymes CAT, SOD and GPX (Table 1).

For catalase, Site 3, the largest symmetric tetrameric junction (617.1 Å^3^), showed strong binding (ΔGbind = −38.82 kcal/mol). The interaction involved symmetry-related hydrogen bonds with H364 and hydrophobic contacts with P368, P391 and M392 across multiple chains (Figure S2C).

Site 4, an interfacial pocket (463.7 Å^3^), exhibited the highest predicted affinity (ΔGbind = −53.08 kcal/mol). BPA formed hydrogen bonds with A44 and S426 and hydrophobic contacts with F286, F297 and P347 (Figure S2B). Both sites map to regions critical for tetramer assembly suggesting the potential to destabilize tetramer assembly.

Site 17 (464.1 Å^3^) displayed strong binding (ΔGbind = −48.69 kcal/mol) featuring hydrogen bonding to W303 and Q442, π-π stacking with H305 and F446 and hydrophobic interactions with V302 and F198 (Figure S2F).

Site 15 (ΔGbind = −44.04 kcal/mol) spans the interface between Chain A and the NADPH-binding domain of Chain C, forming hydrogen bonds with H63, L371 and R388, π-edge interactions with H63 and H395 and hydrophobic contacts with V383 and Q387 (Figure S2E). These sites overlap structurally with the folded NADPH-binding cleft, potentially competing with NADPH binding, which is known to protect catalase from oxidative inactivation.

Site 14 (ΔGbind = −34.55 kcal/mol) involves residues lining the hydrophobic substrate channel, forming hydrogen bonds with S120 and R170, π-π stacking with H175 and hydrophobic contacts with Tyr325 and Phe326 (Figure S2D). This pocket corresponds to the molecular-ruler constriction that selectively admits hydrogen peroxide; ligand binding here could block peroxide access to the heme active site.

For SOD1, all four sites target the dimer interface and regions near the active site. Site 1 (ΔGbind = −59.04 kcal/mol; XP GScore = −6.260 kcal/mol; Figure S3B) showed the strongest predicted affinity and is located at the primary dimer interface (volume = 277.1 Å^3^), with the highest SiteScore (1.040).

BPA formed two hydrogen bonds with V8 and G52, a π-cation interaction with K10 from Chain B, aromatic H-bonds with G52, V8 and V149 from both chains and 50 hydrophobic contacts, primarily with V149, N54 and K10.

Site 2 (ΔGbind = −57.70 kcal/mol; XP GScore = −5.415 kcal/mol; Figure S3C), a large hydrophobic pocket spanning the A/B interface (volume = 284.3 Å^3^), showed a similarly high affinity. BPA formed three hydrogen bonds with K4, S112 and the backbone carbonyl of I114, a π-cation interaction with R116, aromatic H-bonds with K4 and L107 and extensive hydrophobic contacts dominated by I152, M3 and I114. Ligand binding at these dimer-interface and core-packing regions could destabilize the native SOD1 fold.

Sites 3 and 4 are positioned near the electrostatic loop and active site channel. Site 3 (ΔGbind = −31.68 kcal/mol; XP GScore = −3.342 kcal/mol; Figure S3D) is a small surface cavity (volume = 49.0 Å^3^). BPA formed hydrogen bonds with A76 and R80, a π-cation interaction with R80, aromatic H-bonds with H68, D77, E78 and R80 and hydrophobic contacts dominated by V104.

Site 4 (ΔGbind = −36.04 kcal/mol; XP GScore = −4.603 kcal/mol; Figure S3E) formed three hydrogen bonds with H64, T136 and K137, a π-cation interaction with K137, aromatic H-bonds with H64 and T136 and 31 hydrophobic contacts primarily with K137 and P63.

Site 3 engages H68 (a copper ligand) and R80, which are part of the active site’s inner coordination sphere and electrostatic network. Site 4 engages the bridging histidine (H64), which is critical for proton transfer during catalysis and K137, part of the electrostatic loop. Ligand binding in these regions could directly hinder substrate access to the copper or disrupt the electrostatic potential, essential for guiding superoxide.

For GPX, Site 3, a symmetric junction involving all four chains, showed the highest SiteScore (1.052) but did not accommodate BPA binding (Figure S4A).

Site 1 showed the strongest binding affinity (ΔGbind = −49.18 kcal/mol; XP GScore = −3.464 kcal/mol; Figure S4B), representing one of two nearly identical symmetric sites at the A/B and C/D interfaces (SiteScore = 1.193–1.194, volume = 162.2–162.6 Å^3^). BPA formed hydrogen bonds with E83A and S93B, along with hydrophobic contacts involving N84 and V97. The binding was further stabilized by aromatic hydrogen bonds with Q82, S45, E83 and S93.

These interfacial sites involve residues 45–98, which are in the N-terminal domain critical for tetramer assembly and structural stability of GPX1. BPA binding at these symmetric dimer–dimer interfaces could potentially disrupt quaternary structure integrity.

Site 4 demonstrated strong binding (ΔGbind = −46.66 kcal/mol; XP GScore = −3.589 kcal/mol; Figure S4C) at a large cavity (volume = 277.1 Å^3^) spanning multiple chains. BPA formed hydrogen bonds with W160, T143 and Q82, along with π-π edge-to-face stacking with W160 and π-cation interaction with R179. Extensive hydrophobic contacts were dominated by W160 and Y147.

This site involves residues 76–180, encompassing regions near the C-terminal domain where the selenocysteine catalytic residue (Sec47) and its surrounding microenvironment are located. BPA binding here could potentially interfere with the catalytic machinery and substrate access to the active site.

Site 7 (ΔGbind = −39.11 kcal/mol; XP GScore = −4.043 kcal/mol; Figure S4D), positioned at the B/C interface (volume = 81.6 Å^3^), formed hydrogen bonds with P138 and N129, with hydrophobic contacts predominantly from P132C and P138B. This site represents another dimer–dimer interface that could affect tetramer stability.

Site 11 (ΔGbind = −23.02 kcal/mol; Figure S4E) showed moderate binding at a single-chain cavity (volume = 127.3 Å^3^) on Chain A. BPA formed hydrogen bonds with S151 and N77, π-π stacking with H81 and hydrophobic contacts primarily with I149. This site involves residues 76–151, overlapping with regions important for maintaining the structural integrity of the GPX1 monomer.

## 4. Discussion

Endocrine disruptors with estrogenic activity, such as bisphenol A (BPA), can bind to receptors on sperm, thus altering their function [53]. Environmental pollutants have multifaceted impacts on reproductive health and ecosystems, as highlighted by Piscopo et al. [54]. We established a comprehensive in vitro test system to evaluate whether BPA interferes with sperm function, membrane phospholipid composition, and sperm amino acid profile. Furthermore, we evaluated the effect of *Myrtus communis* L. essential oil on the prevention of these alterations, as well as its role in BPA redistribution potential with respect to bisphenol A free in spermatozoa and the culture medium. Moreover, we studied the bioavailability of the essential oil compounds within spermatozoa, as well as its protective effect against BPA-induced oxidative stress.

Our results showed that a 100 µM concentration of BPA significantly reduced sperm motility and viability after one hour of incubation. Although the 100 µM concentration used in this study is higher than BPA levels typically detected in human biological fluids or environmental samples, which are generally reported in the nanomolar range [55,56], such concentrations are commonly used in in vitro toxicological studies to elucidate oxidative and metabolic disturbances within a limited experimental timeframe. Similar effects were observed in vivo in rats [57] and mice [58], particularly regarding motility. However, data from in vitro exposures in mammalian sperm remain limited. To our knowledge, only the study by Md Saidur Rahman et al. reported that in vitro exposure of mouse sperm to 100 µM BPA for 6 h resulted in a decrease in sperm parameters [59]. Therefore, the decrease in motility observed after exposure to 100 µM BPA was correlated with a reduction in the level of cardiolipin, a key phospholipid involved in proper mitochondrial functioning [60]. Interestingly, we observed an increase in phosphatidylcholine (PC), suggesting that BPA disrupts phosphatidylcholine biosynthesis; this observation highlights the crucial role of phospholipid homeostasis in maintaining normal cellular functions [61].

In our study, BPA exposure resulted in a decrease in several amino acids, including arginine. The detailed changes are summarized in Table 4.

The accumulation of free BPA in spermatozoa observed in our study confirms previous findings that BPA, as a lipophilic endocrine-disrupting compound, can readily penetrate cell membranes and accumulate within reproductive cells [70]. The absence of free BPA in the culture medium of spermatozoa exposed solely to BPA suggests rapid uptake and retention within the cells, which may interfere with sperm function and viability [71]. Such intracellular accumulation has been associated with oxidative stress, reduced motility, and DNA damage in mammalian sperm [72]. Interestingly, co-treatment with myrtle essential oil resulted in a dose-dependent reduction in free BPA levels in spermatozoa, accompanied by a corresponding increase in the culture medium at the highest concentration (5 µL/mL). This indicates that components of the essential oil may facilitate either the efflux or the sequestration of BPA from sperm cells, thereby reducing intracellular exposure. Essential oils are known to exhibit membrane-modulating and antioxidant properties that can mitigate xenobiotic accumulation and oxidative damage [73]. The protective effect observed here is consistent with studies showing that phytochemicals can reduce BPA-induced cytotoxicity in reproductive cells by promoting BPA redistribution and limiting cellular uptake [74,75].

Interestingly, alpha-pinene is detected in sperm at a dose of 1 µL/mL. This is explained by the ability of this chemotype to penetrate the cell and exert a local protective effect, especially since the best motility is observed at this dose, thus confirming the direct effect of alpha-pinene. Furthermore, alpha-pinene has a high affinity for biological lipid compartments due to its hydrophobic structure [76]. Pharmacokinetic studies show that alpha-pinene, after absorption or exposure, can accumulate in body lipid compartments before being metabolized and excreted. In experimental models, alpha-pinene has been detected in blood and other lipid-rich tissues, and its urinary metabolites can be used as biomarkers of exposure [77]. Furthermore, the lack of additional protective effects at the high dose (5 µL/mL) could reflect a phenomenon of membrane saturation or equilibrium of lipophilic compounds. Indeed, excessive integration of lipophilic molecules into membranes can alter membrane homeostasis, which could indirectly affect motility, viability, or other sperm functions, especially under stressful conditions [78]. The non-monotonic detection of alpha-pinene, observed only at the intermediate dose (1 µL/mL) and absent at both lower (0.5 µL/mL) and higher (5 µL/mL) doses, may reflect threshold-dependent membrane partitioning. At low concentrations, insufficient compounds are available to penetrate and accumulate within spermatozoa, whereas at high concentrations, membrane saturation or disruption could limit intracellular retention. Additionally, analytical detection limits relative to the sample volume extracted could contribute to the apparent absence at extreme doses. This phenomenon highlights the complex interplay between lipophilic compound uptake, membrane dynamics, and bioavailability within sperm cells.

Exposure to BPA induced significant oxidative stress in spermatozoa, as evidenced by increased MDA levels, reflecting enhanced lipid peroxidation, and by the reduction in both enzymatic (SOD, CAT-like, GPx-like) and non-enzymatic (thiols) antioxidant defenses. Oxidative stress is known to impair sperm membrane integrity and cellular function, leading to reduced sperm quality and fertility [79]. These results are consistent with previous studies showing that BPA generates reactive oxygen species (ROS) in male reproductive cells, leading to membrane damage, enzyme inactivation, and impaired sperm function [80,81]. Treatment with EOMC effectively attenuated BPA-induced oxidative stress, significantly reducing MDA levels and restoring the activity of antioxidant systems, including SOD, CAT-like and GPx-like activities, as well as thiol content. This protective effect may be attributed to the antioxidant properties of monoterpenes, such as α-pinene, present in EOMC, which can scavenge ROS and stabilize cellular membranes [82]. These findings support the potential of EOMC as a natural protective agent against BPA-induced oxidative damage in spermatozoa [83]. In general, the redistribution effect of EOMC observed in this study appears primarily linked to its antioxidant properties, which neutralize BPA-induced oxidative stress and preserve sperm cellular integrity. This protective action may help maintain membrane stability and support the activity of endogenous antioxidant enzymes such as SOD, CAT-like and GPX-like, thereby contributing to the recovery of sperm motility and viability. Furthermore, the reduction in intracellular BPA concentrations in sperm, coupled with the increase detected in the culture medium, suggests that EOMC could also influence membrane dynamics and facilitate BPA redistribution or elimination. It should be noted that several non-mutually exclusive mechanisms may underline this redistribution effect. These include passive redistribution mediated by EOMC-induced changes in membrane fluidity and permeability, competitive displacement of BPA from intracellular lipid compartments by highly lipophilic terpene constituents of EOMC, and modulation of membrane transporter activity. While these hypotheses are consistent with the observed data and physicochemical properties of EOMC compounds, they remain speculative and require experimental validation. However, a direct chemical interaction between BPA and EOMC compounds cannot be confirmed based on the current results and requires further investigation.

It is important to note that the antioxidant enzyme activities measured in the present study should be interpreted as GPx-like and CAT-like activities rather than strictly enzyme-specific activities. Indeed, spermatozoa are known to contain high levels of perox-iredoxins (PRDX), which represent a major component of the antioxidant defense system in these cells and play a central role in the detoxification of hydrogen peroxide (H_2_O_2_), as well as other reactive oxygen and nitrogen species, including peroxynitrite (ONOO^−^) and organic hydroperoxides, thereby contributing to redox regulation. In particular, PRDX6 has been shown to play a key role in protecting spermatozoa against both ROS and RNS [84,85]. Importantly, PRDX are highly sensitive to oxidative stress and can undergo inactivation under conditions of excessive reactive oxygen species production [86]. Therefore, it is plausible that BPA-induced oxidative stress may also target PRDX, contributing to the overall impairment of antioxidant defenses observed in this study. This may partly explain the decrease in GPx-like, and CAT-like activities reported here, as these measurements likely reflect the combined activity of multiple antioxidant systems.

Our findings demonstrate that exposure to bisphenol A (BPA) may induce a significant reduction in antioxidant defense capacity in spermatozoa, highlighting a disruption of cellular redox homeostasis. Molecular docking simulations predict thermodynamically favorable binding of BPA to multiple structurally and functionally relevant sites across all three antioxidant enzymes. For catalase, BPA was predicted to bind within the NADPH-binding pocket and along the hydrogen peroxide access channel. Since tightly bound NADPH protects catalase from oxidative inactivation and supports efficient H_2_O_2_ decomposition, occupation or distortion of this region by BPA would be expected to increase susceptibility to inactivation and limit substrate access to the heme center. In SOD1, BPA predicted favorable binding near the electrostatic loop and active-site channel, regions that guide superoxide anions toward the copper catalytic center and stabilize the catalytic geometry. For GPx1, BPA was predicted to bind close to the selenocysteine-centered catalytic microenvironment, which may reduce GPx catalytic efficiency and cellular peroxide processing.

The present study has several limitations that should be acknowledged. First, the number of biological replicates was limited (*n* = 3 per group), mainly due to the complexity of the experimental design and the resource-intensive nature of the analyses performed, including LC–MS-based lipidomic, metabolomics, and enzymatic assays. Although this sample size may reduce statistical power, the inclusion of three technical replicates for each measurement ensured the reproducibility and consistency of the obtained results. Nevertheless, future studies with larger sample sizes are warranted to strengthen the statistical robustness of the findings. Second, the in silico analysis was restricted to the interaction between bisphenol A (BPA) and antioxidant enzymes (CAT, SOD1, and GPX1). No molecular docking simulations were conducted for the major bioactive constituents of *Myrtus communis* essential oil (α-pinene, 1,8-cineole, and d-limonene). Therefore, although the experimental data demonstrates a protective effect of EOMC on antioxidant enzyme activity, the precise molecular mechanisms underlying this effect remain to be elucidated. Future investigations integrating molecular docking and dynamic simulations are needed to better characterize the interactions between EOMC compounds and antioxidant enzymes.

## 5. Conclusions

BPA induces severe sperm dysfunction in vitro, characterized by impaired sperm motility and viability, oxidative stress, and alterations in membrane lipid and amino acid composition. EOMC exerts dose-dependent protective effects, restoring sperm function, reducing intracellular BPA accumulation, and mitigating oxidative and metabolic disturbances at low and medium concentrations. However, higher doses may induce biphasic responses or toxic effects. Molecular modeling confirms BPA’s potential to interact directly with and impair antioxidant defense system. It should be noted that this study was performed using an in vitro model, which may not fully replicate in vivo conditions. These findings highlight the therapeutic potential of EOMC as a natural agent to mitigate BPA-induced sperm toxicity and underscore the importance of dosage optimization for effective protection. These results provide new insights into the underlying mechanisms of BPA toxicity and its impact on male fertility, while also offering a potentially beneficial solution for reproductive medicine.

## Figures and Tables

**Table 1:** Docking results for BPA across antioxidant enzymes.

Enzyme	Site ID	SiteScore	XP GScore	MMGBSA ΔG_bind_
CAT	4	1.099	−5.27	−53.08
CAT	17	1.091	−6.06	−48.69
CAT	15	1.077	−5.87	−44.04
CAT	3	1.201	−6.20	−38.82
CAT	14	1.177	−5.08	−34.55
SOD	1	1.040	−6.260	−59.04
SOD	2	0.820	−5.415	−57.70
SOD	4	0.593	−4.603	−36.04
SOD	3	0.534	−3.342	−31.68
GPX	1	1.193	−3.464	−49.18
GPX	4	0.906	−3.589	−46.66
GPX	7	0.781	−4.043	−39.11
GPX	11	0.658	−2.570	−23.02

**Table 2:** Effect of the incubation of epididymal sperm cells with EOMC for 1 h at different concentrations, followed by co-incubation for further 1 h with BPA on profile amino acids.

Amino Acids (ng/mL)	Control	BPA	EOMC (0.5 µL/mL) + BPA	EOMC (1 µL/mL) + BPA	EOMC (5 µL/mL) + BPA
**Arginine**	0.08 ± 0.05	0 ± 0 ^a^	0 ± 0 ^a^	0.04 ± 0.01	0 ± 0 ^a^
**Histidine**	0.45 ± 0.21	0 ± 0 ^a^	0 ± 0 ^a^	0 ± 0 ^a^	0 ± 0 ^a^
**Methionine**	0 ± 0	0 ± 0	0 ± 0	0 ± 0	0 ± 0
**Alanine**	0 ± 0	0 ± 0	0.98 ± 0.04 ^ab^	1.10 ± 0.45 ^ab^	0 ± 0
**Phenylalanine**	0.18 ± 0.06	0 ± 0 ^a^	0.06 ± 0.02 ^a^	0.10 ± 0.03 ^b^	0 ± 0 ^a^
**Glutamine**	0 ± 0	0 ± 0	0 ± 0	0 ± 0	0 ± 0
**Glycine**	0 ± 0	0 ± 0	0 ± 0	0 ± 0	0 ± 0
**Aspartic Acid**	0 ± 0	0 ± 0	0 ± 0	0 ± 0	0 ± 0
**Valine**	0.148 ± 0.05	0.130 ± 0.003	0.138 ± 0.05	0.195 ± 0.05	0.05 ± 0.03
**Serine**	0.84 ± 0.10	0 ± 0 ^a^	0 ± 0 ^a^	0 ± 0 ^a^	0 ± 0 ^a^
**Proline**	0.24 ± 0.11	0.13 ± 0.05	0.17 ± 0.07	0.18 ± 0.08	0.14 ± 0.11
**Lysine**	0.12 ± 0.06	0 ± 0 ^a^	0.013 ± 0.01^a^	0.12 ± 0.03^b^	0.08 ± 0.01 ^ab^
**Tryptophan**	0 ± 0	0 ± 0	0 ± 0	0 ± 0	0 ± 0
**Threonine**	0 ± 0	0 ± 0	0 ± 0	0 ± 0	0 ± 0
**Tyrosine**	0 ± 0	0 ± 0	0 ± 0	0 ± 0	0 ± 0
**Asparagine**	0 ± 0	0 ± 0	0 ± 0	0 ± 0	0 ± 0
**Leucine**	0.49 ± 0.01	0.22 ± 0.03	0.35 ± 0.16	0.63 ± 0.07	0.22 ± 0.01

Control: negative control; BPA: Bisphenol A; EOMC + BPA: *Myrtus communis* essential oil (0.5, 1, and 5 µL/mL) with BPA. ^a^ *p* < 0.05 compared to control group and ^b^ *p* < 0.05 compared to BPA group (ANOVA test). *n* = 3 independent biological replicates per group, with 3 technical replicates per assay. Values of 0 ± 0 indicate that the amino acid was below the detection limit in all replicates for that group. Significant differences (letters) are relative to groups with detectable values.

**Table 3:** Effect of the incubation of epididymal sperm cells with EOMC for 1 h at different concentrations, followed by co-incubation for further 1 h with BPA on sperm oxidative stress.

	Control	BPA	EOMC (0.5 µL/mL) + BPA	EOMC (1 µL/mL) + BPA	EOMC (5 µL/mL) + BPA
**MDA** (nmol/mg protein)	8.70 ± 0.51	12.95 ± 1.26 ^a^	11.16 ± 0.62	8.42 ± 1.30 ^b^	13.95 ± 0.8 ^a^
**CAT** (nmol H_2_O_2_/min/mg protein)	4.02 ± 0.96	0.15 ± 0.003 ^a^	0.79 ± 0.50	3.28 ± 2.88	0.42 ± 0.18
**SOD-like activity** (U/mg protein)	5.11 ± 0.42	0.90 ± 0.035 ^a^	1.92 ± 0.69 ^ab^	4.82 ± 0.40	0.93 ± 0.027 ^ab^
**GPx-like activity** (nmol GSH/min/mg protein)	0.17 ± 0.007	0.02 ± 0.016 ^a^	0.10 ± 0.001 ^ab^	0.12 ± 0.01 ^ab^	0.02 ± 0.01 ^a^
**Groupement thiols** (umol/mg protein)	53.83 ± 18.52	17.94 ± 6.88 ^a^	46.27 ± 4.80 ^b^	48.61 ± 6.64 ^b^	20.52 ± 9.85 ^a^

Control: negative control; BPA: Bisphenol A; EOMC + BPA: *Myrtus communis* essential oil (0.5, 1, and 5 µL/mL) with BPA. ^a^ *p* < 0.05 compared to control group and ^b^ *p* < 0.05 compared to BPA group (ANOVA test). *n* = 3 independent biological replicates per group, with 3 technical replicates per assay.

**Table 4:** Summary of amino acids decreased after BPA exposure, their known functional roles in male reproduction, and supporting references.

Amino Acid	Change After BPA Exposure	Known Functional Role/References
Arginine	Decreased	Essential for spermatogenesis. For example, a diet deficient in arginine can lead to multinucleated giant cells as well as impaired spermatogenesis in men [62], whereas supplementation has been reported to improve sperm number and motility [63,64,65].
Phenylalanine	Decreased	Supports sperm motility and contributes to the activation of key signaling pathways involved in reproductive function [66].
Proline	Decreased	According to Yen and Curran (2021) [67], inactivation of proline dehydrogenase is relatively benign; however, disruption of ALH-6 induces premature reproductive aging [67].
Valine	Decreased	According to Dong et al. (2016), valine supplementation improves sperm parameters, seminal plasma composition, and offspring outcomes [68].
Leucine	Decreased	Stimulates protein synthesis and supports spermatogenesis and male fertility [69].

## Data Availability

The data that support the findings of this study are available upon request from the corresponding author. The data is not publicly available due to privacy or ethical restrictions.

## Supplementary Materials

The following supporting information can be downloaded at: https://www.mdpi.com/article/10.3390/antiox15050536/s1, Figure S1: Validation of 3D model quality for antioxidant enzymes. Structural validation results for (A) superoxide dismutase [Cu-Zn], (B) catalase and (C) glutathione peroxidase 1; Figure S2: Molecular docking of bisphenol A (BPA) to catalase binding sites; Figure S3: Molecular docking of bisphenol A (BPA) to superoxide dismutase [Cu-Zn] binding sites; Figure S4: Molecular docking of bisphenol A (BPA) to glutathione peroxidase 1 binding sites; Table S1: Chemical composition of the essential oil of *Myrtus communis* L. leaves as determined by GC–MS analysis.
