# Supplementary material for: Protective Effects of Myrtus communis Essential Oil Against Bisphenol A-Induced Sperm Dysfunction: Insights from Lipidomic, Amino Acid Profiling, Oxidative Stress and Molecular Docking
Source: Antioxidants (Basel). 2026 Apr 24;15(5):536. doi: 10.3390/antiox15050536 (PMC13203262; doi:10.3390/antiox15050536)
Supplement: Supplementary file 1 [file antioxidants-15-00536-s001.zip › antioxidants-4191924-supplementary.pdf]

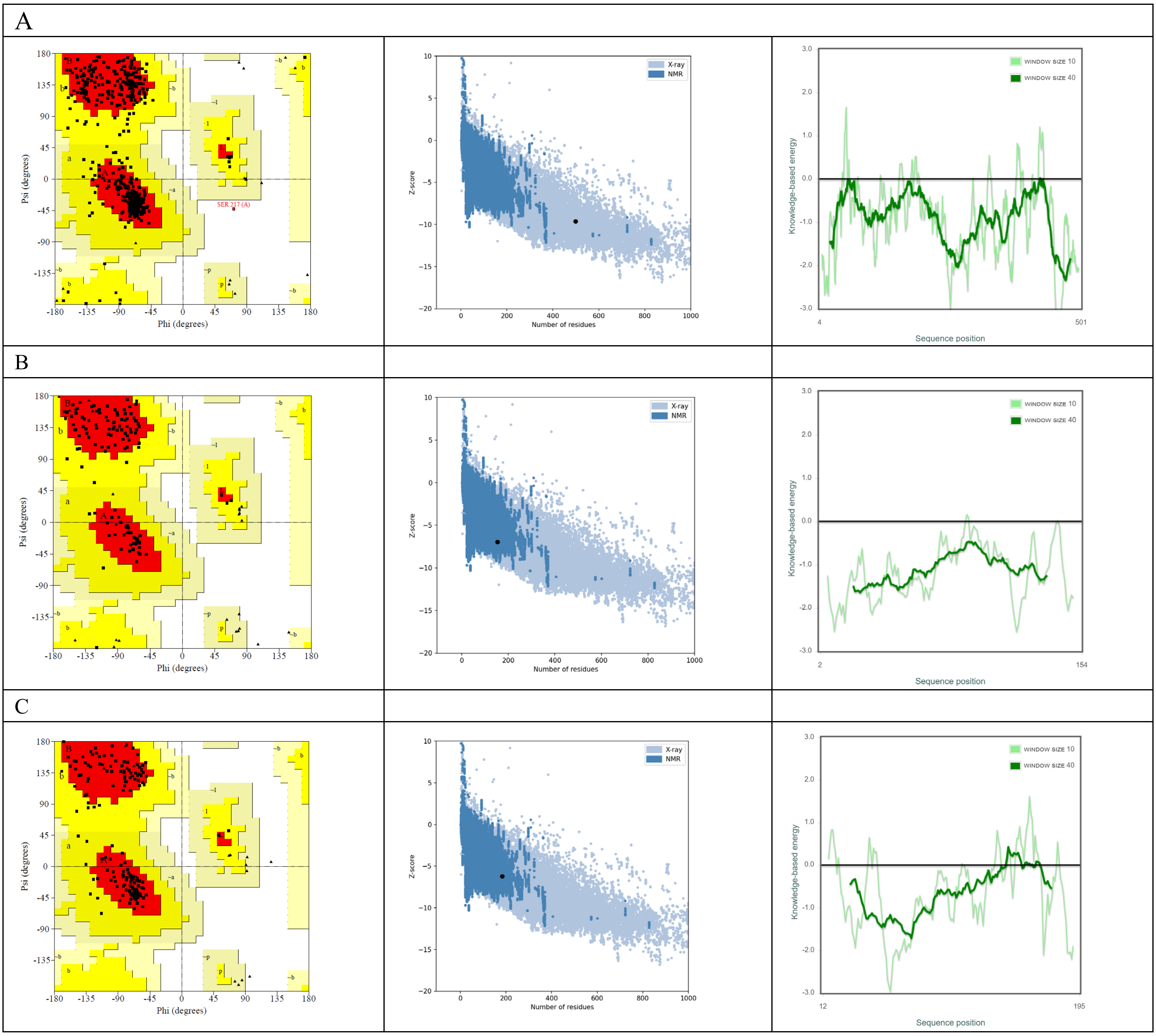

Figure S1. Validation of 3D model quality for antioxidant enzymes. Structural validation results for (A) superoxide dismutase [Cu-Zn], (B) catalase and (C) glutathione peroxidase 1. Left column: Ramachandran plots generated using SAVES-PROCHECK show the distribution of phi-psi angles. Middle column: ProSA-web overall model quality plots showing z-scores (black spot) within the range of experimental structures based on number of residues. Right column: ProSA-web local model quality plots showing knowledge-based energy values along the sequence; negative or near-zero energy values correspond to error-free regions of the structure.

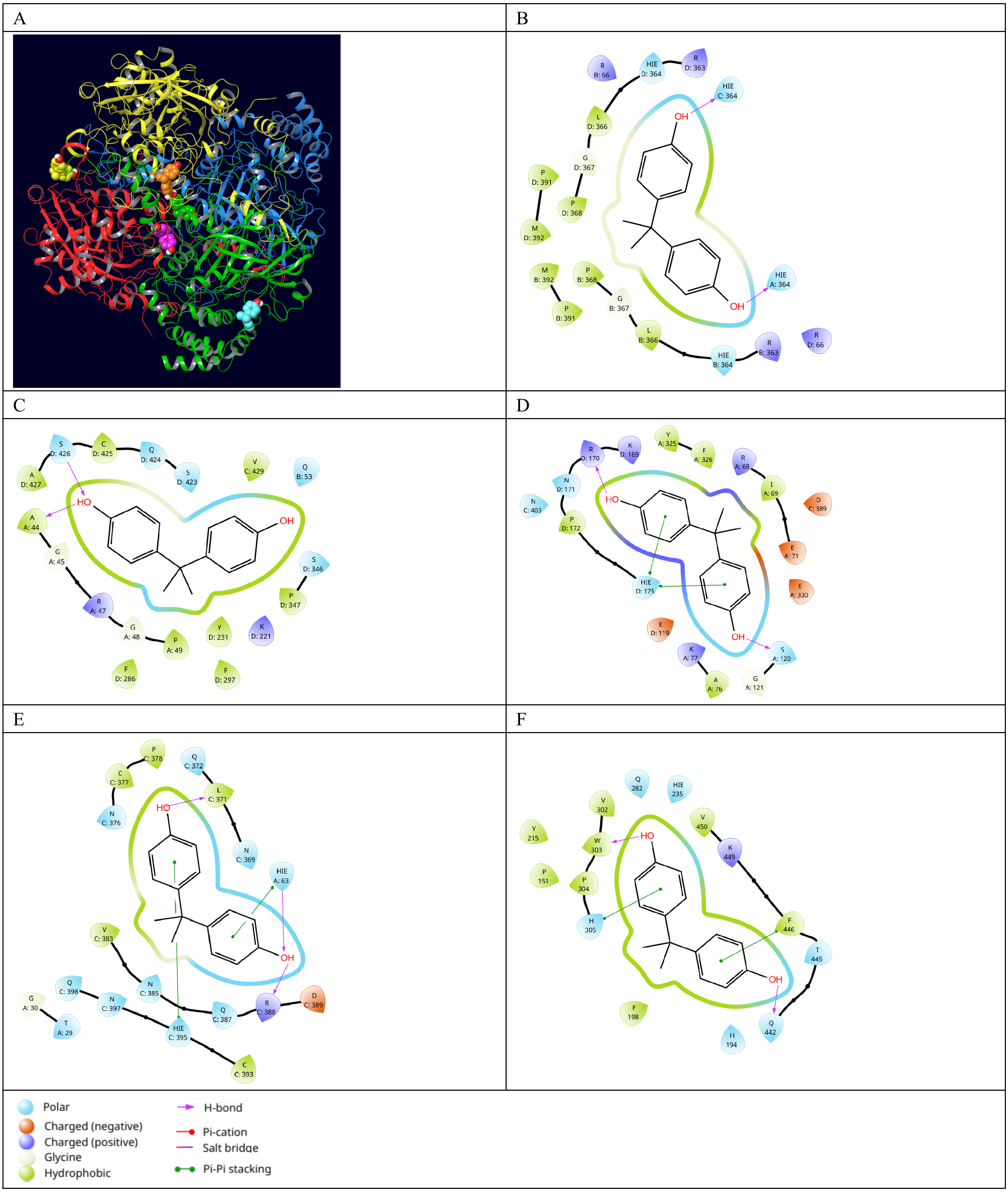

Figure S2. Molecular docking of bisphenol A (BPA) to catalase binding sites. (A) Spatial distribution of the five representative binding sites on the catalase tetramer structure: site 3 (green), site 4 (magenta), site 14 (cyan), site 15 (orange) and site 17 (yellow). (B-F) Two-dimensional interaction diagrams showing the binding mode and key molecular interactions of BPA at each catalase binding site: (B) site 3, (C) site 4, (D) site 14, (E) site 15 and (F) site 17.

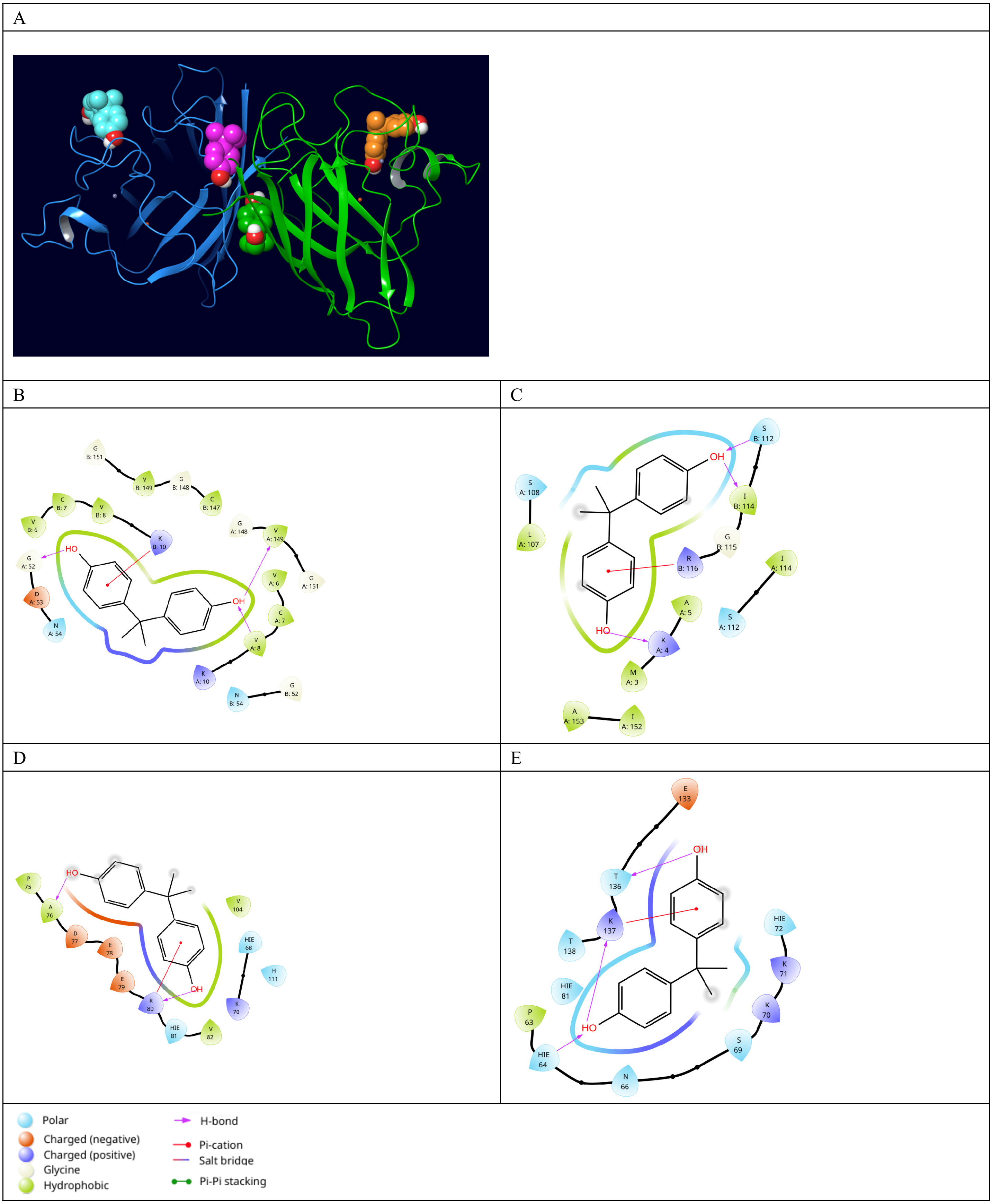

Figure S3. Molecular docking of bisphenol A (BPA) to superoxide dismutase [Cu-Zn] binding sites. (A) Spatial distribution of the four representative binding sites on the SOD1 dimer structure: site 1 (green), site 2 (magenta), site 3 (cyan) and site 4 (yellow). Cu^{2+} and Zn^{2+} metal ions are shown as spheres. (B-E) Two-dimensional interaction diagrams showing the binding mode and key molecular interactions of BPA at each SOD1 binding site: (B) site 1, (C) site 2, (D) site 3 and (E) site 4.

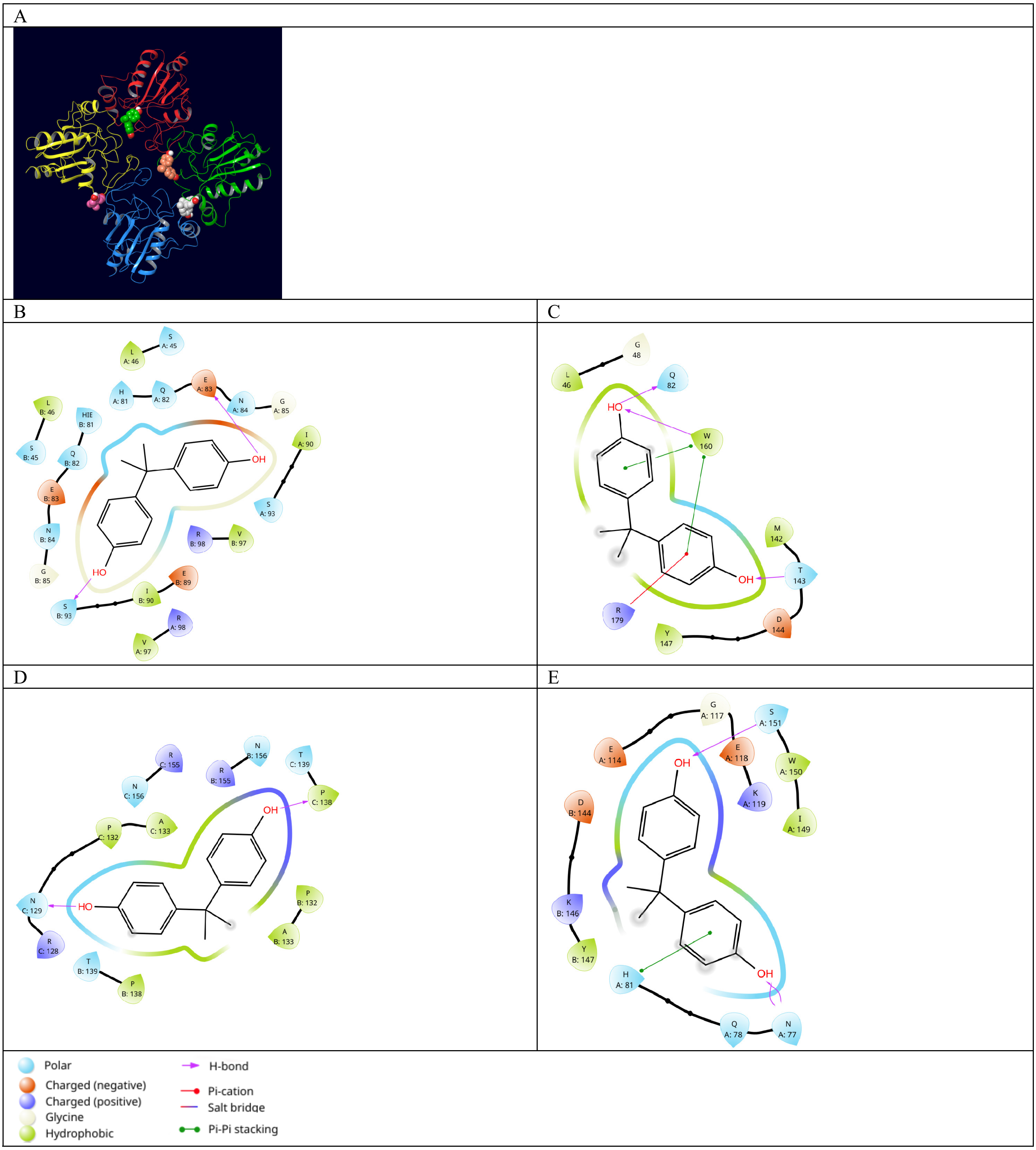

Figure S4. Molecular docking of bisphenol A (BPA) to glutathione peroxidase 1 binding sites. (A) Spatial distribution of the three representative binding sites on the GPX1 tetramer structure: site 1 (green), site 4 (magenta), site 7 (cyan) and site 11 (orange). Selenocysteine residues are highlighted. (B-D) Two-dimensional interaction diagrams showing the binding mode and key molecular interactions of BPA at each GPX1 binding site: (B) site 1, (C) site 4, (D) site 7 and (E) site 11.

Table S1: Chemical composition of the essential oil of *Myrtus communis* L. leaves as determined by GC–MS analysis.

Peak	Compounds	Relative content (%)	RT (min)
1	Alpha. -Thujene	0.618	6.009
2	Alpha. -Pinene	59.749	6.197
3	Beta. -Pinene	0.410	7.111
4	Butanoic acid, 2-methyl-, 2-methylpropyl ester	0.260	7.674
5	l-Phellandrene	0.450	7.739
6	Delta.3-Carene	0.768	7.879
7	p-Cymene	2.297	8.214
8	dl-Limonene	7.020	8.337
9	1,8-Cineole	18.651	8.393
10	gamma-Terpinene	0.808	9.030
11	Alpha-Terpinolene	1.164	9.746
12	Linalool	1.666	10.001
13	Butanoic acid, 2-methyl-, 2-methylbutyl ester	0.435	10.093
14	Alpha. Terpineol	1.241	12.203
15	Linalyl acetate	0.624	13.676
16	α-Terpinenyl acetate	0.447	15.815
17	Geranyl acetate	1.911	16.515
18	Methyleugenol	0.804	16.996
19	Caryophyllene	0.676	17.413

Retention time (RT, min)
